# Supplementary material for: V–, U–, L– or W–shaped economic recovery after Covid-19: Insights from an Agent Based Model
Source: PLoS One. 2021 Mar 2;16(3):e0247823. doi: 10.1371/journal.pone.0247823 (PMC7924750; doi:10.1371/journal.pone.0247823)
Supplement: Attachment — Submitted filename: rebuttal_letter.pdf [file pone.0247823.s001.pdf]

Response to Reviewers for manuscript
"V–, U–, L– or W–shaped recovery after Covid-19: Insights from an Agent Based Model"

We wish to thank all the three referees for their careful reading of our manuscript and for their very useful suggestions to improve it. Below we provide point-by-point answers to their comments.

Reviewer 1

1. The Results section should be presented at the end, after describing the model and all approximations. In this section, the authors describe the parameters of the model before describing the model itself.

   Response: The description of the model has now been moved to before the "Results" section for better readability.

2. 99-106 and table 1 shows the empirical parameters of the model. However, their significance is not substantiated.

   Response: The discussion around the empirical parameters and baseline values for some of the important variables like inflation has now been placed in the section where the details of the model have been provided.

   The parameters enumerated in Table 1 have been chosen to generate a baseline scenario such that the economy finds itself in the Full-Employment phase in the language of [1–3].

   We found that the qualitative behavior of the model, which is the focus of our work, does not change radically upon changing the precise value of most parameters within a reasonable range. Because our aim is not to calibrate the model or to provide quantitative predictions, we believe that this choice is satisfactory for our purposes. We follow here the methodological manifesto that we have proposed in our previous work [1].

3. The Politics section offers different scenarios for overcoming the crisis, but without prior formalization of the mathematical model, it is impossible to understand these scenarios.

   Response: All relevant details of the model are now presented before the results are presented.

4. ABM models - depend on random values. Therefore, the simulation results may be unstable. It is not clear from Figures 4 and 5 this is the result of the work of an ensemble of models or a single simulation. The authors do not provide an analysis of the sensitivity and stability of the model from changes in various factors.

   Response: Figures 4 and 5 represent the results of a single simulation. This has also been clarified in the text of the manuscript. While we present only a single representative simulation in Figures 4 and 5, we have repeated each simulation

multiple times and have found that all our results are robust. We note this in lines 193-198 of the manuscript.

In these figures, for the baseline scenarios — without any policy — we have used parameters for the shock $\Delta\zeta/\zeta$ and $\Delta c/c$ that push the economy into a state of permanent crisis (the L-shaped scenario). The robustness of these parameters has been verified through the phase diagram presented in Figure 2.

We also add that the phase diagrams in Figures 2 and 3 have been constructed using the results from 500 independent simulations for each pair $\Delta\zeta/\zeta$-$\Delta c/c$.

5. The Model section should be moved to the beginning of the article. In this section it is expedient to formalize the model in the form of the scheme of interaction of various components-blocks of system

   Response: All details of the model are now present before any of the results are presented to help the reader better understand the model. The interaction scheme of the model, in the baseline version used here, is extremely simple: one representative household interacts with N heterogeneous firms (via prices, wages, and dividends) and both interact in a trivial way with a central bank (via savings and credit lines). The model is so simple in this respect that we have the impression that a scheme would not add much to the readers' understanding.

6. It would be expedient to justify the choice of values of empirical parameters of the model to the characteristics of a particular country.

   Response: Within this work, we have taken the route of using an Agent-based model as a "scenario generator" to focus the attention on general aspects of post-Covid recovery. As stated above (and in the introduction of the paper), our goal is not to calibrate the model to provide quantitative predictions, but to assess the different qualitative scenarios that can emerge from the non-linear feedback present in the economy.

   Hence, parametrizing the model for a particular country is beyond the scope of this work. Certain aspects of the model are borne out in the data: for instance, the annualized inflation rate sets the time scale in our model. Its value has been set to be similar to the (pre-Covid) inflation rate in the Eurozone area.

   It should also be noted that some of our qualitative conclusions match those of more quantitative ABMs, for example the recent OECD ABM calibrated on UK data which also reveals the need of extended policy support and the existence of tipping points beyond which the economy does not recover spontaneously, see [4] (Ref. [24] in the revised manuscript).

7. It would be interesting to compare the effectiveness of the strategies of different countries in comparison with the results of the model.

   Response: Only a qualitative comparison between countries is possible at this time within our model. A detailed analysis and comparison between countries would require adding more details to the model such as a government sector, followed by a calibration exercise for a particular country.

   Nevertheless, the results presented in this work point towards the risks of removing aid too quickly to firms and households. We elaborate on this in the concluding section of the paper.

Reviewer 2

1. The short title is better than long title that creates confusion.

Response: The longer title now includes the word "economic" to make the main thesis of the paper clearer.

2. The study is interesting with the aim to suggest guidelines for economic recovery after COVID-19 pandemic crisis and shock of lockdowns.

3. Introduction has to provide more theoretical background about COVID-19 and risk factors current and future in society to know the problem for health and economies (see suggested readings that have to be read and used in the text).

   Response: The introduction now includes the references that the reviewer has suggested. Changes have been made to reflect the challenges and risk factors to both health and economies in the introductory section.

4. The study is based on simulations and without empirical evidence. Of course, the recovery depends on countries. I suggest to clarify better research setting because countries are different . . . a recovery in Italy is different from a recovery in Sweden because of different structural indicators. . . . for instance, Italy has a high public debt and a similar shock generates different effect than other countries. Moreover, also the lockdown generates different effects according to the duration. . . longer lockdown generates a higher deterioration of economic systems than short one (see new literature). How these aspects are considered in the model? The contraction of some economies is higher than others.

   Response: Within the current model, we are only able to perform a qualitative comparison between countries. Comparing the situation of different countries would require a more detailed model followed by a calibration exercise for the country in the question.

   Nonetheless, the results presented in this work point towards the risks of removing aid too quickly to firms and households. We elaborate on this in the concluding section of the paper as well.

   Finally, as mentioned in the main text, the duration of the shock is indeed a key parameter of the model. The difference between short and longer lock-downs has been described along with efficient policies has been described and presented in Figures 4 and 5.

5. In short, there are manifold variables to control and I suggest whenever possible to discriminate the design of the model for countries that have some similarities, such as Spain and Italy, Germany and France, Scandinavian countries etc. to be more consistent with the reality and reduce the heterogeenity between countries and provide more reliable policy implications. As you know the applications of policies generates different effects according to the countries and the structure of their economies. . . I would like to know how it is possible a match between these different shapes L, W, etc. of recovery and different countries, and policy implications. . . . I know it is necessary a time consuming revision, but authors can provide more indications to provide a main contribution for political economy of growth post COVID-19.

   Response: We have explained above and in the introduction that the scope of our work is not to provide quantitative predictions for a specific country. We believe that our model is way too simple to be properly calibrated on empirical data for a given country, and our goal is not to provide precise quantitative predictions for policy making.

   Our aim, following the methodological manifesto presented in [1], is to analyze the possible scenario that are generated by some nonlinear feedback channels that, we

believe, are present in the real economy and are also at work in our model. We show indeed that the model can generate a variety of scenarios. This allows us to explore how the different scenarios are impacted by a given policy or by a change in a given parameter.

Yet, it is comforting to see that some of our qualitative conclusions match those of more quantitative ABMs, for example the recent OECD ABM calibrated on UK data which also reveals the need of extended policy support and the existence of tipping points beyond which the economy does not recover spontaneously [4](Ref. [24] in the revised manuscript).

Hence we believe that our study can help shape intuition into basic economics mechanisms, but the study suggested by the referee is way to ambitious given the present status of the model and we believe that it definitely goes beyond the scope of the present work.

Yet, the policy mix we study, though limited, suggests some important pathways for limiting the damage from Covid-19. We have added some references that reviewer 2 suggested in the introduction, which address this point.

6. Conclusion has to present better manifold limitations of this study for the complexity of factors and heterogeneity of countries and in particular for the methods of inquiry based on simulations done in a computer lab.

   Response: We have expanded upon the limitations of the present study in the concluding section of the paper.

   We reiterate that the primary objective of the present work is to demonstrate how ABMs can be used as “scenario generators” to test policy prescriptions under “what-if” conditions. This is a limited ambition, which is nevertheless of value in our radically uncertain world. Our results are thus of a general nature and the *exact* details would necessarily depend on the unique characteristics of any particular country. This limitation has now been addressed in the concluding section of the paper.

7. Suggested readings of relevant papers that have to be read and all inserted in the text and references to improve the study.

   - Leach, M., MacGregor, H., Scoones, I., Wilkinson, A. 2021Post-pandemic transformations: How and why COVID-19 requires us to rethink development, World Development 138,105233
   - Coccia M., 2020. National lockdown to cope with COVID-19 pandemic: effects (contradictory) on public health and (negative) on economic system, Working Paper CocciaLab n. 56D/2020, CNR – National Research Council of Italy. Available at Research Square, DOI is: 10.21203/rs.3.rs-115665/v1
   - Abuselidze, G., Slobodianyk, A. 2021 Pandeconomic crisis and its impact on small open economies: A case study of COVID-19 Advances in Intelligent Systems and Computing, 1258 AISC, pp. 718-728
   - Coccia M. 2020. An index to quantify environmental risk of exposure to future epidemics of the COVID-19 and similar viral agents: Theory and Practice. Environmental Research, Article number 110155, DOI: 10.1016/j.envres.2020.110155
   - Yoshino, N., Hendriyetty, N. 2020 The COVID-19 Crisis: Policy Recommendations for Japan. Economists' Voice , 17(1),20200017
   - Iuga, I.C., Mihalciuc, A. 2020Major crises of the XXIst century and impact on economic growth. Sustainability (Switzerland), 12(22),9373, pp. 1-20

   Response: The aforementioned references have now been added to the manuscript.

Reviewer-3

The article is well written and provides interesting insights about the response of the economy to the pandemic due to COVID-19. In general, the authors did a good job explaining the purpose of the study, and described properly the instruments they used to develop the study. I have some minor comments for the authors to be addressed before the study can be accepted for publication.

1. I suggest including some literature in the introduction (Lines 40-44) about using ABM to understand the impacts of COVID. For example, please review the following article. https://doi.org/10.1016/j.ssci.2020.105022

   Response: This reference has now been added in the introduction section of the manuscript.

2. Line 204: The authors could explain better why they selected 2 policies. Are there different policies that simulate the same effects from the policies that the authors used in the study in the literature? If so, explain why other policies are not selected.

   Response: The two policies that we have considered here have been discussed in the literature or in the commentariat. Extending direct credit support to firms has been done to various degrees in all developed economies of the world. We have hence considered such a policy and examined how the duration of the policy, for robust recovery, must extend beyond well after the pandemic is behind us.

   The second policy, i.e. helicopter money, has also been discussed in various places and advanced countries have tried to supplement household wages through various channels, with the most important example being that of the US through the CARES Act (2020). Of course one could consider many other policies, but the main scope of our work is to present a set of qualitative scenarios and, most importantly, to show that our model can produce some interesting non-trivial behavior via non-linear feedback. The code has been made public and the model can be easily modified by the reader. Our hope is to motivate other groups to use the model to investigate other policies.

   For example, a policy that we have not considered here is for the government to keep paying wages directly to citizens. While this would require an explicit public sector to be integrated within the model, the effects would be equivalent to an increase in the bankruptcy parameter Θ, as considered here.

3. I suggest to the authors to include in the study some discussion about how the results were validated and verified. If not, please also state that clearly.

   Response: For the phase diagrams in Figures 2 and 3, 500 independent simulations were done for each pair of values $\Delta\zeta/\zeta$ — $\Delta c/c$. Following this, for figures 4 and 5 we have chosen parameters so that the baseline scenario (without policy) corresponds to an L-shaped recovery. While we only present a representative simulation in Figures 4 and 5, we have verified that our results are qualitatively robust by performing multiple simulations with different seeds for our random number generator.

   In interest of clarity, we have added that figures 4 and 5 correspond to a single representative run of the model. We also checked that modifying the basic parameters given in Table 1 within reasonable ranges does not affect substantially the results.

4. I would also recommend to the authors to include the word economy in the title.

   Response: We have updated the title of the paper, by replacing recovery with economic recovery.

References

1. Gualdi S, Tarzia M, Zamponi F, Bouchaud JP. Tipping points in macroeconomic agent-based models. Journal of Economic Dynamics and Control. 2015;50:29–61. doi:10.1016/j.jedc.2014.08.003.

2. Gualdi S, Tarzia M, Zamponi F, Bouchaud Jp. Monetary policy and dark corners in a stylized agent-based model. Journal of Economic Interaction and Coordination. 2017;12(3):507–537. doi:10.1007/s11403-016-0174-z.

3. Bouchaud JP, Gualdi S, Tarzia M, Zamponi F. Optimal inflation target: insights from an agent-based model; 2018. *Economics Discussion Papers, No 2017-64. Kiel Institute for the World Economy.* Available from: `http://www.economics-ejournal.org/economics/discussionpapers/2017-64`.

4. Barnes S, Hillman R, Wharf G, MacDonald D. Impact of COVID-19 on Corporate Fragility: Insights from a new calibrated firm-level Corporate Agent-Based (CAB) Model; 2020. OECD, Working Paper, 2020.
